# The effects of gamma glutamyl cysteinyl ethyl ester in traumatically brain injured rats post incident: insight into the role of glutathione elevation in oxidative stress

**DOI:** 10.3389/fnins.2026.1892068

**Published:** 2026-09-23

**Authors:** Jenna Shoemaker, Patrick G. Sullivan, Andrea Sebastian, Tanea T. Reed

**Affiliations:** 1 Department of Chemistry, Eastern Kentucky University, Richmond, KY, United States; 2 Spinal Cord and Brain Injury Research Center, University of Kentucky, Lexington, KY, United States

**Keywords:** antioxidant, gamma glutamyl ethyl ester, glutathione, oxidative stress, traumatic brain injury

## Abstract

Traumatic brain injury (TBI) is a spontaneous event in which the brain sustains primary and secondary injuries. There are more than 2.3 million traumatic brain injuries occurring annually in the United States and exponentially more worldwide. These injuries may be mild, moderate, or severe based on intensity of impact. The damage caused by TBI results not only from the initial injury, but also from secondary damage due to DNA damage, protein oxidation, and oxidative stress which can impact overall brain function. Oxidative stress is the phenomenon in which there is an increase in the level of reactive oxygen and nitrogen species. A decrease in overall antioxidant capacity, which can lead to a loss of protein function is observed. Antioxidant therapies have been evaluated as potential treatments for traumatic brain injury. Glutathione, a potent antioxidant is depleted in traumatic brain injury. Gamma-glutamyl cysteinyl ethyl ester (GCEE), a glutathione mimetic, has been used as an antioxidant to alleviate oxidative stress. Currently, there are no approved treatments for TBI to prevent secondary injury post incident, but immediate medical attention is recommended as a delay in treatment as elevations in secondary consequences are exhibited. This study aims to determine if GCEE is neuroprotective against moderate TBI at an extended time point, 90 min as positive outcomes in reduction of oxidative stress markers were observed at lower time point (15, 30, and 60 min). Results from this work will provide insight into the alleviation of oxidative stress markers via antioxidant treatment of moderate traumatic brain injury at an expanded time point.

## 1 Introduction

Traumatic brain injury (TBI) is a spontaneous phenomenon that occurs when an external force makes an impact on the brain. This can be through blunt force or piercing through the skull. Examples of incidents that can cause a traumatic brain injury are gunshot wounds, car accidents, and falling and hitting the head. Traumatic brain injury happens in two separate phases: primary and secondary. Primary injury occurs when the impact force causes damage to the axonal tissue. Consequences of primary injury can include the disruption of cell membranes and increased cellular permeability (Chen et al., 2023; Moppett, 2007). Molecules that are typically not allowed to pass through the membrane may now enter causing damage that may be irreversible. While primary injury can cause biological deficits, secondary injury causes most of the main complications in traumatic brain injury. Secondary injury is the cascade of molecules that occurs anywhere from ninety minutes up to months after the initial insult. Secondary injury can result from the initial injury itself, processes following the initial injury, or extracerebral events (Chesnut, 2004). Consequences of secondary injury include protein oxidation, DNA damage, and oxidative stress (Moppett, 2007). All have been well documented in neurodegenerative disorders and traumatic brain injury (Balakrishnan et al., 2026; Lai et al., 2026; Mohammed et al., 2026; Senousy et al., 2026; de Macedo Filho et al., 2024). There are a variety of risk factors that are known to be associated with poor outcomes in TBI. These factors can be divided into two different categories: factors that are associated with the time of the primary TBI and factors that are associated with secondary traumatic injury. One risk factor is that TBIs that penetrate the brain are more detrimental than external forces.

Traumatic brain injury is one of the leading causes of injury worldwide. In 2023, 69,000 traumatic brain injury-related deaths occurred in the United States. Over 200,000 annual hospitalizations and rising medical expenses contributed to non-fatal TBI cases and the impacted healthcare systems (CDC, 2026; Miller et al., 2021; Pavlov et al., 2019). Approximately 5.3 million Americans have a disability from a traumatic brain injury (Hicks et al., 2026). A percentage of those cases were sustained via combat injuries from military service (Dismuke-Greer et al., 2025). TBI can vary ranging from mild to severe. Mild traumatic brain injury can have a variety of symptoms and are usually associated with concussions. Associated symptoms for mild TBI can include headaches, nausea, irritability, dizziness, and lethargy (Peterson et al., 2019). Moderate TBIs may be demonstrated by cognitive, physical, and behavioral changes attributed to the incident including loss of consciousness. Severe traumatic brain injury can cause more life-threatening symptoms due to the acuteness of the injury itself. These symptoms can include but are not limited to slurred speech, a decline in coordination, seizures, difficulty recognizing people and faces, and loss of consciousness (CDC, 2024). With fatality and a change in the quality of life, there is also a financial cost for trying to increase the quality of life and decrease morbidity rates in patients with a traumatic brain injury. In 2019, 4.2 trillion dollars were spent on the cost of these injuries including medical care, insurance companies, work loss, and quality of life improvements (Peterson et al., 2019). Approximately 60% of the total costs were among adults aged 25–64 which compromise the majority of the workforce (Peterson et al., 2019). While symptoms can vary, the consequences of ignoring a traumatic brain injury and not seeking medical attention can be fatal and drastically decrease quality of life. Therefore, the families impacted by traumatic brain injury in this age group must incur income loss as well as the stress of caring for an ailing individual and paying medical bills. Since there is no current treatment for traumatic brain injury, this illness can be devastating for any family. With the number of traumatic brain injuries increasing every year, it is imperative to understand how traumatic brain injury can affect quality of life. These injuries greatly impact the family members as they now serve as caretakers which can impact their own personal and mental health (Leibach et al., 2014; Nonterah et al., 2013). A traumatic brain injury can happen at any age. Younger and older persons typically are at higher risk due to falls. While men have a higher risk for TBI, women have a risk factor in severity as they are more likely to have swelling in the brain and hypertension as well (Moppett, 2007). Children who are intentionally injured, pedestrians, and cyclists have a higher morbidity rate (Moppett, 2007). Young adult males have the highest risk for a significant traumatic brain injury, and this is typically due to motor vehicle accidents and alcohol use (Moppett, 2007). Many individuals with TBI also are at higher risk simply because they feel they do not need to seek medical attention and proper evaluation after an incident with potential for a secondary injury.

Oxidative stress is a mechanism where increased reactive nitrogen (RNS) and oxygen species (RNS) react in the brain. If the reactive oxygen and nitrogen species increase in excess, they will be toxic and destroy proteins required for proper brain function. As the brain is vulnerable to lipid peroxidation due to its high amounts of polyunsaturated fatty acids, it is at high risk for having lower levels of antioxidants to combat oxidative stress. This can have dire consequences when the antioxidant to ROS and RNS ratio favor the reactive species. Glutathione, a potent antioxidant, has lower production during oxidative stress (Bizzozero et al., 2007; Lok et al., 2011). Oxidative stress biomarkers can be used to determine the extent of the traumatic brain injury that has occurred. Protein carbonyls may be used to detect protein oxidation, while 3-nitrotyosine (Figure 1) and 4-hydroxynenal (Figure 2), may be used to detect protein nitration and lipid peroxidation, respectively. These three biomarkers of oxidative stress are increased in all classifications of traumatic brain injury, mild (Muneer et al., 2013), moderate (Butterfield and Reed, 2016; Henderson et al., 2016) and severe (Bayir et al., 2002; Seifman et al., 2008).

**Figure 1:**
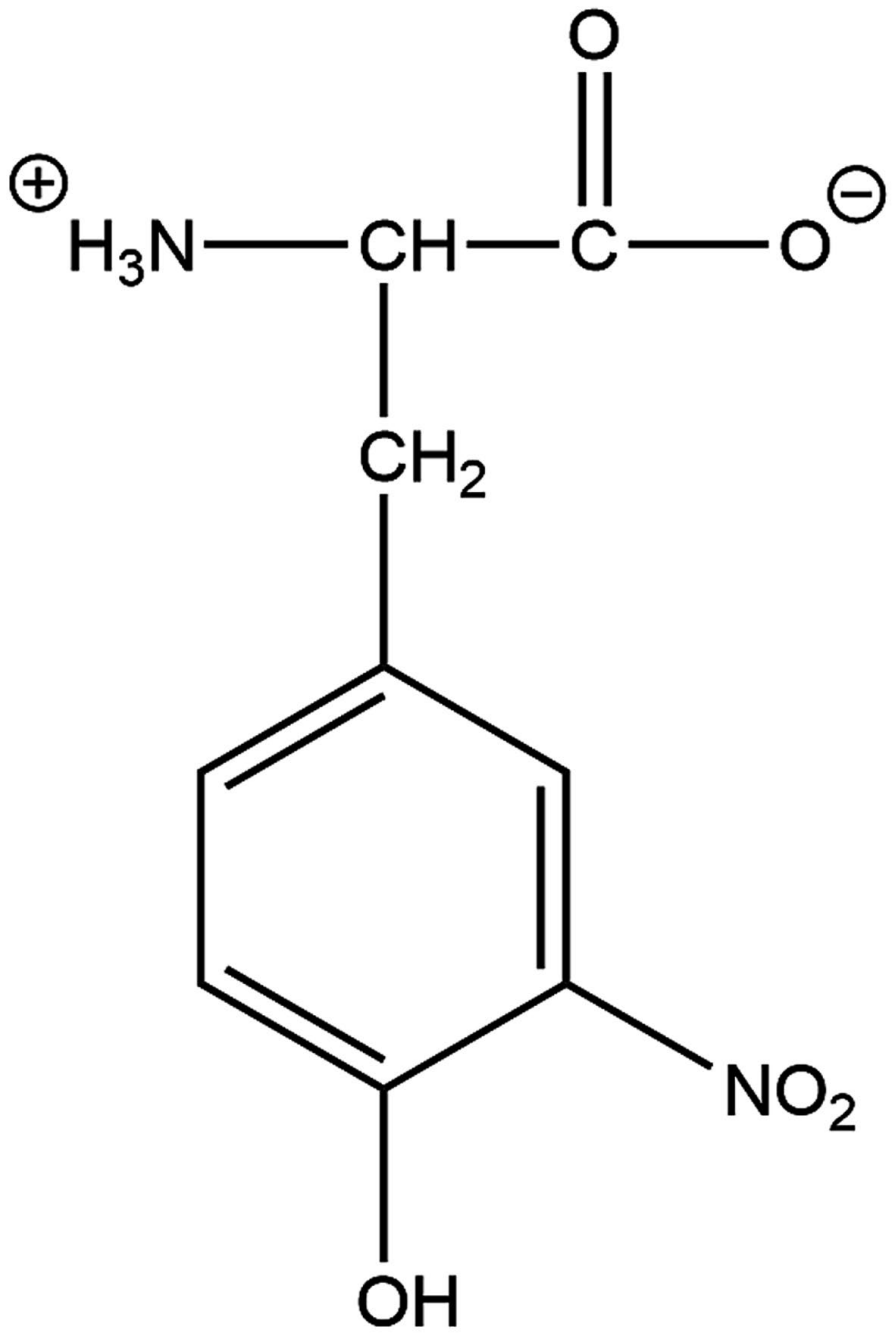
Structure of 3-nitrotyrosine.

**Figure 2:**
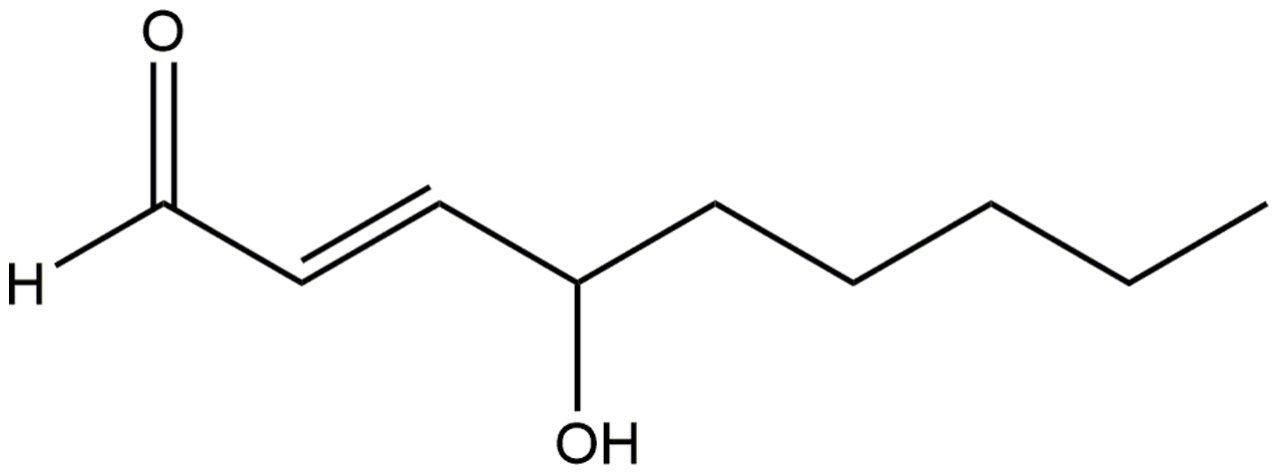
Structure of 4-hydroxynonenal (HNE).

Diagnostic tools in hospital settings are not typically sufficient in determining the severity of a traumatic brain injury. Parameters such as brain pH, intracranial pressure, and temperature are limited at best in diagnosing TBI (Guingab-Cagmat et al., 2013). Imaging methods including computer tomography (CAT) scans and magnetic resonance imaging (MRI) are only able to capture the state of the brain in one noticeably brief moment in time (Guingab-Cagmat et al., 2013). This brain imaging captured at a specific time point may not be the full extent of the injury and can only attest to the levels of damage that are developing at that time. There are new strategies for examining blood biomarkers as identification and evaluation of TBI status (Decouty-Perez et al., 2026; Elkhodair et al., 2026; Nazeer and Murtaza, 2026; Quadri et al., 2026; Wongsripuemtet et al., 2026). These are exciting discoveries that may elucidate the mystique of this disorder and provide more insight into therapeutic strategies.

There is no current treatment for traumatic brain injury. However, as oxidative stress is increased in traumatic brain injury, antioxidant therapies are being studied (Davis et al., 2022; Davis and Vemuganti, 2022). As glutathione is depleted and glutathione related enzymes are decreased traumatic brain injury (Bayir et al., 2002; Ansari et al., 2008), methods to increase glutathione is an ideal research focus for plausible antioxidant treatment (Giustarini et al., 2023). Gamma glutamyl cysteinyl ethyl ester (GCEE) is a molecule known to have antioxidant properties (Drake et al., 2002). GCEE, as depicted in Figure 3, has also been demonstrated to show a reduction in oxidative stress in the brain as a glutathione mimetic, thereby GCEE allows the reuptake of glutathione into the brain. Furthermore, this allows glutathione to bind with reactive species instead of allowing the reactive species to bind to molecules in the brain needed to function properly. In previous studies, GCEE has successfully reduced oxidative stress in moderate traumatically brain-injured rats when administered up to sixty minutes post injury (Butterfield and Reed, 2016).

**Figure 3:**
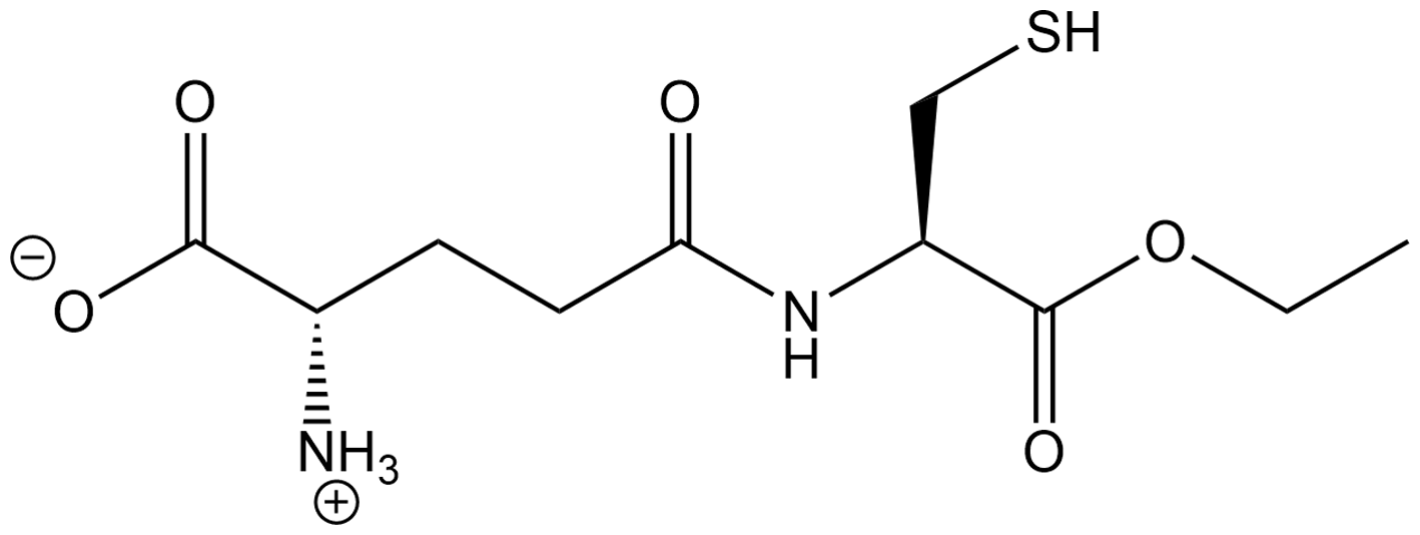
Structure of gamma glutamyl cysteinyl ethyl ester (GCEE).

Unfortunately medical care may not be administered within an hour due to extending waiting time, hospital staff availability, and travel times to medical and/or specialized trauma centers if needed (Nyce et al., 2021), therefore effective delayed treatment strategies should be investigated. This study aims to evaluate the levels of oxidative stress biomarkers in rats given a moderate TBI and treated with GCEE at 90 min post-injury to determine neuroprotection against oxidative stress. Results from this project will provide insight for strategies for administering treatment post-injury in regard to more realistic wait times.

## 2 Materials and methods

All chemicals, unless indicated, were obtained from Sigma Aldrich (St. Louis, MO, USA) and were of the highest purity. GCEE was purchased from Bachem (Torrance, CA, USA). All water used in this procedure was distilled and deionized. Brain tissue samples were stored at −80 °C until used. Once homogenized in a buffer solution, samples were stored at −20 °C until used.

### 2.1 Surgical methods

The surgical procedures and drug administration protocols described below are in compliance with and have been approved by the University of Kentucky Institutional Animal Care and Use Committee (protocol code: D16-00217; date of approval: 1 August 2016) and are consistent with all animal care procedures established by the U.S. Public Health and Service Policy on Humane Care and Use of Laboratory Animals.

In this study, 24 Wistar adult male rats were used, each weighing 300–350 g. Isoflurane (3.0%) was used to anesthetize the animals. The rats were shaved and then placed in a stereotaxic frame (David Kopf Instruments, Tujunga, CA, USA). A craniotomy and a 1.5 mm contusion was introduced for the moderate traumatic brain as described by Sullivan et al. (2002). Sham animals were excluded from the injury but received a craniotomy (*n* = 6). Six animals were naïve (no injury and no craniotomy). Post craniotomy, each Wistar rat had a 4 mm dental cement seal placed on the craniotomy site. The rats were then placed on warming mats to prevent hypothermia until they regained consciousness. The rats were given 150 mg/kg body weight of GCEE 90 min post injury (n = 6). Injections were given intraperitoneally based on prior research in which improvement with post-neurosteroidal treatment was observed (Lok et al., 2011; Djebaili et al., 2005). Another group was given saline at 90 min post injury, with a concentration of 150 mg/kg body weight (*n* = 6). Concentrations for GCEE and saline were determined from previous studies (Reed et al., 2009). All rats were then sacrificed 24 h post injury with immediate removal and freezing of the whole brain at −80 °C. Brain tissue was collected at this time. The time period of 24 h was chosen due to having maximum decrease in glutathione levels as well as enzymatic activity (Reed et al., 2009). Peak levels of protein carbonyls, 3-nitrotyrosine, and 4-hydroxynonenal are observed 24–48 h post injury (Ansari et al., 2008). However it should be noted that significant changes in oxidative stress levels in protein carbonyls were observed in five minutes post injury (Smith et al., 1994) and thirty minutes for 3-nitrotyrosine and 4-hydronenal levels (Hall et al., 1993). Therefore, the timepoint for GCEE administration and sample collection occurs after significant oxidative stress has occurred. Brain samples were then sonicated and placed in 10 mM HEPES buffer (pH 7.4) containing 137 mM NaCl, 4.6 mM KCl, 1.1 mM KH_2_PO_4_, 0.1 mM EDTA, and 0.6 mM MgSO_4_ along with protease inhibitors: leupeptin (0.5 mg/mL), pepstatin (0.7 μg/mL), type II S soybean trypsin inhibitor (0.5 μg/mL), and phenylmethylsulfonyl fluoride (PMSF; 40 μg/mL). After homogenation, samples were then centrifuged at 14,000 × g for 10 min to remove all cellular debris that could potentially cause matrix effects within the sample. Protein concentration in the supernatant was determined by the BCA protein assay (Pierce, Rockford, IL, USA). Supernatant was then decanted from the centrifuged samples and stored for future determination of enzymatic activity.

### 2.2 Measurement of protein carbonyls

Protein carbonyl measurements were taken 24 h post sacrifice. Five microliters of sample was derivatized with 10 mM 2,4-dinitrophenylhydrazine (DNPH) in the presence of 5 μL 12% sodium dodecyl sulfate for 20 min at room temperature. The samples were then neutralized with 7.5 μL neutralization solution (2 M Tris in 30% glycerol). Derivatized samples (250 ng) were loaded onto a nitrocellulose membrane with a slot blot apparatus in duplicate. The blot was then blocked with 3% bovine serum albumin (BSA) and incubated with rabbit polyclonal anti-DNPH antibody (1:100 dilution) for 2 h. The blot was washed in wash blot buffer (10 mM Tris–HCl, pH 7.5, 150 mM NaCl, 0.05% Tween 20) three times before incubation with a secondary antibody (goat anti-rabbit antibody conjugated with alkaline phosphatase). The blot was washed again and then developed using SigmaFast tablets (Sigma Aldrich), and densiometric levels were quantified in Scion Image software.

### 2.3 Measurement of 3-nitrotyrosine

Three nitrotyrosine measurements were taken 24 h post sacrifice. Five microliters of sample was incubated with 5 μL Laemmli buffer (0.125 M Tris base, pH 6.8, 4% (v/v) sodium dodecyl sulfate (SDS), and 20% (v/v) glycerol) for 20 min at room temperature. The resulting samples (250 ng) were loaded per well in the slot blot apparatus containing a nitrocellulose membrane under pressure. Each sample was loaded in duplicate. After the sample was loaded, the membrane was blocked with 3% (w/v) BSA in phosphate- buffered saline containing 0.01% (w/v) sodium azide and 0.2% (v/v) Tween 20 (PBST) for 1 h and then incubated with a 1:2000 dilution of anti-3-nitrotyrosine polyclonal antibody in PBST for 2 h. After primary antibody incubation, the membrane was carefully washed three times for 5 min each. A secondary antibody of anti-rabbit IgG alkaline phosphatase in PBST (1:3000 dilution) was added to the membrane for 1 h. The membrane was again washed three times in PBST and developed using SigmaFast BCIP/NBT (5-bromo-4-chloro-3-indolyl phosphate/nitro blue tetrazolium) tablets (Sigma). Blots were dried, scanned in Adobe Photoshop (San Jose, CA), and quantitated in Scion Image software.

### 2.4 Measure of 4-hydroxynonenal

Four hydroxynonenal measurements were taken 24 h post sacrifice. Five microliters of sample was incubated with 5 μL Laemmli buffer (0.125 M Tris base, pH 6.8, 4% (v/v) sodium dodecyl sulfate (SDS), and 20% (v/v) glycerol) for 20 min at room temperature. The resulting samples (250 ng) were loaded per well in the slot blot apparatus containing a nitrocellulose membrane under pressure. Each sample was loaded in duplicate. After the sample was loaded, the membrane was blocked with 3% (w/v) BSA in phosphate- buffered saline containing 0.01% (w/v) sodium azide and 0.2% (v/v) Tween 20 (PBST) for 1 h and then incubated with a 1:5000 dilution of anti-HNE polyclonal antibody in PBST for 2 h. After primary antibody incubation, the membrane was carefully washed three times for 5 min each. A secondary antibody of anti-rabbit IgG alkaline phosphatase in PBST (1:3,000 dilution) was added to the membrane for 1 h. The membrane was again washed three times in PBST and developed using SigmaFast BCIP/NBT (5-bromo-4-chloro-3-indolyl phosphate/nitro blue tetrazolium) tablets (Sigma). Blots were dried, scanned in Adobe Photoshop (San Jose, CA), and quantitated in Scion Image software.

## 3 Results

Results for GCEE at 90 min showed that animals treated with GCEE 90 min post injury had lower levels of protein carbonylation than animals treated with saline at 90 min in Figure 4. This is a statistically significant finding as the *p* value is <0.05.

**Figure 4:**
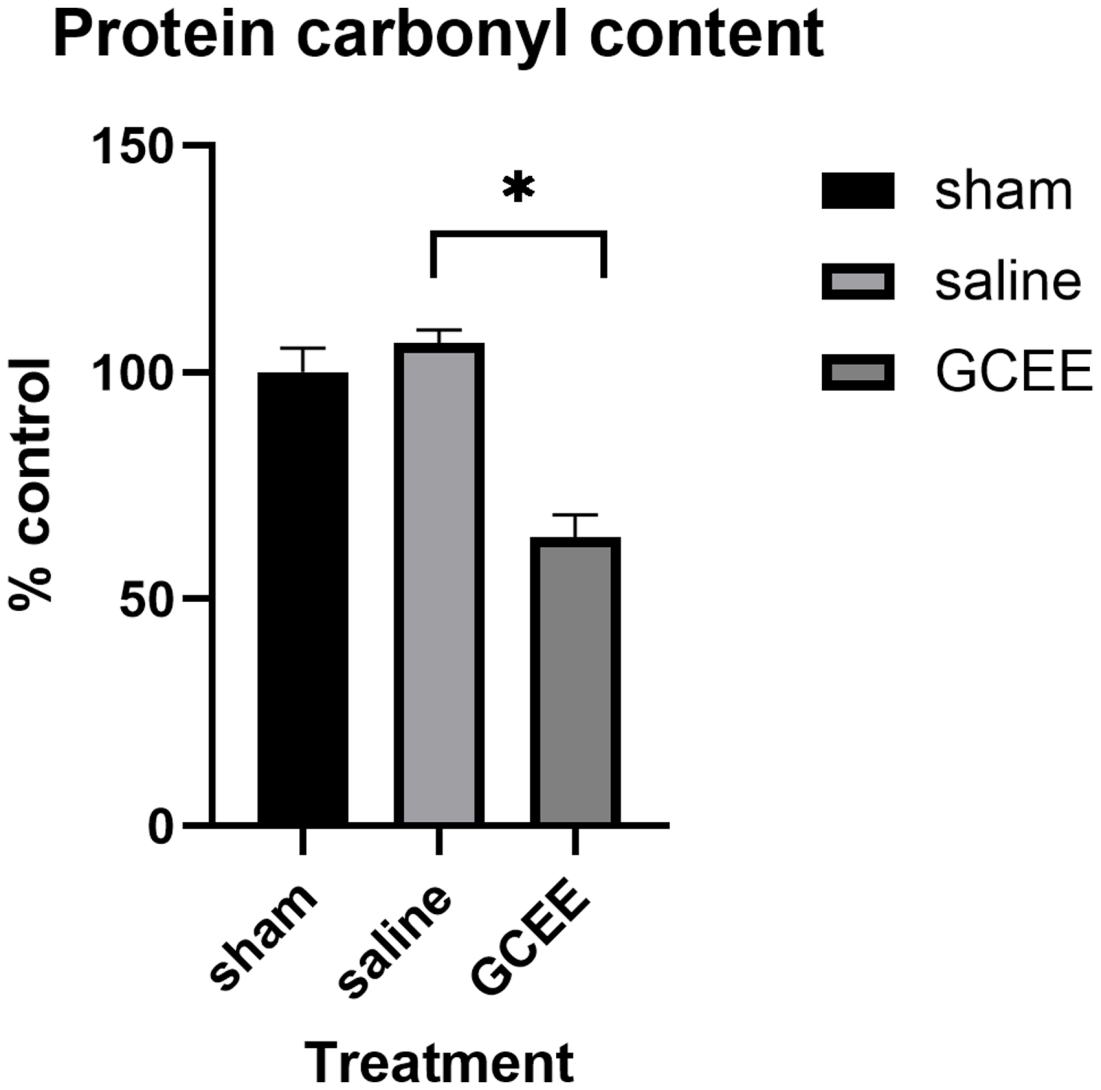
Protein carbonyl content for TBI rats treated via GCEE treatment 90 min post injury. Percentage ± SEM. *N* = 6. *p value <0.005.

Figure 5 shows that animals treated with GCEE at 90 min post injury displayed lower levels of 3-nitrotyrosine than the animals that were treated with saline at 90 min. This indicates that the treatment of GCEE for 90 min is statistically significant in reducing nitrosative stress.

**Figure 5:**
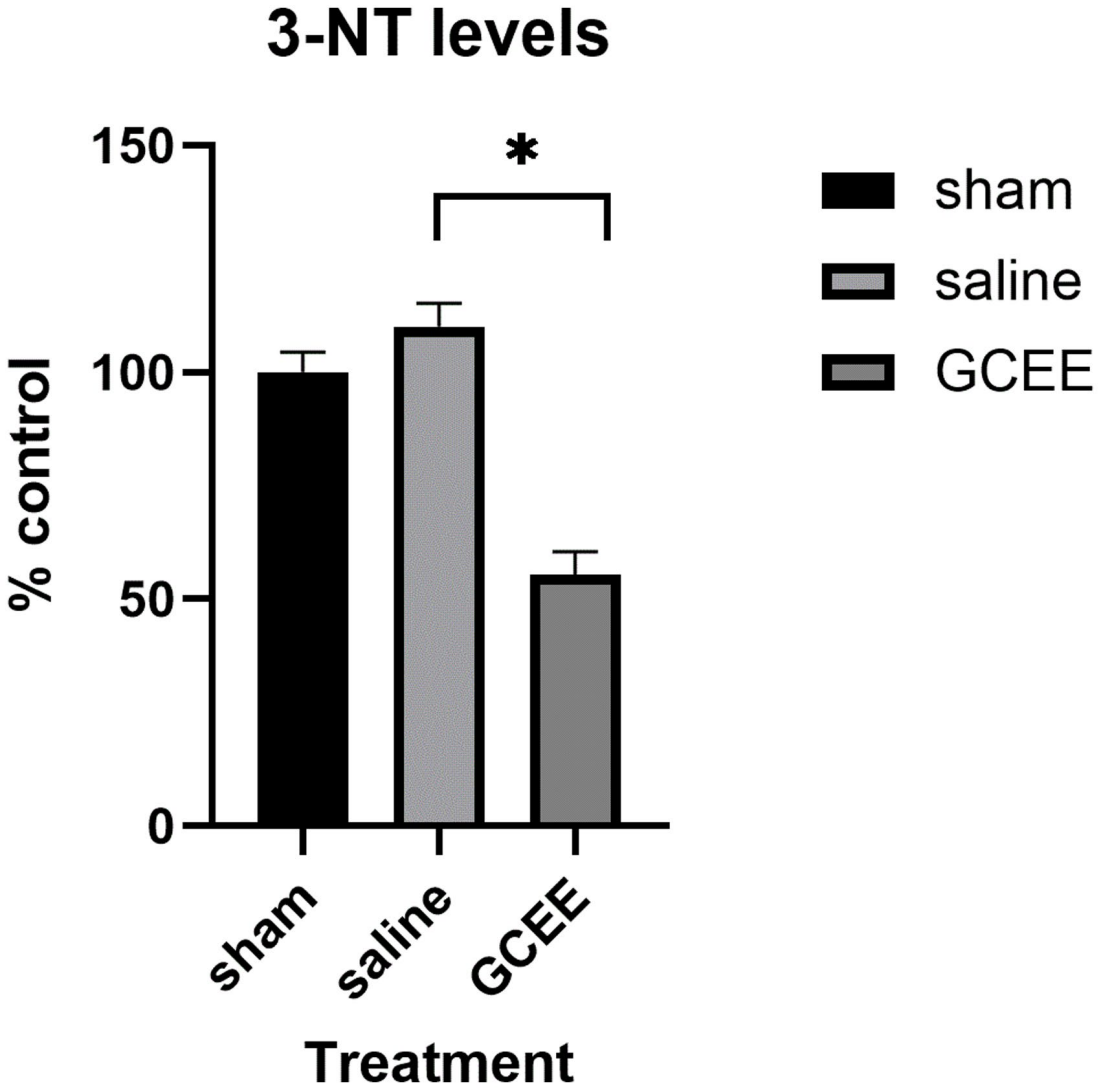
3-Nitrotyrosine levels in TBI rats treated with GCEE 90 min post injury. Percentage ± SEM. *N* = 6. *p value < 0.005.

Animals treated with GCEE 90 min after the initial injury showed lower levels of 4-hydroxynonenal than animals treated with saline 90 min after the initial injury however these were not statistically significant as demonstrated in Figure 6.

**Figure 6:**
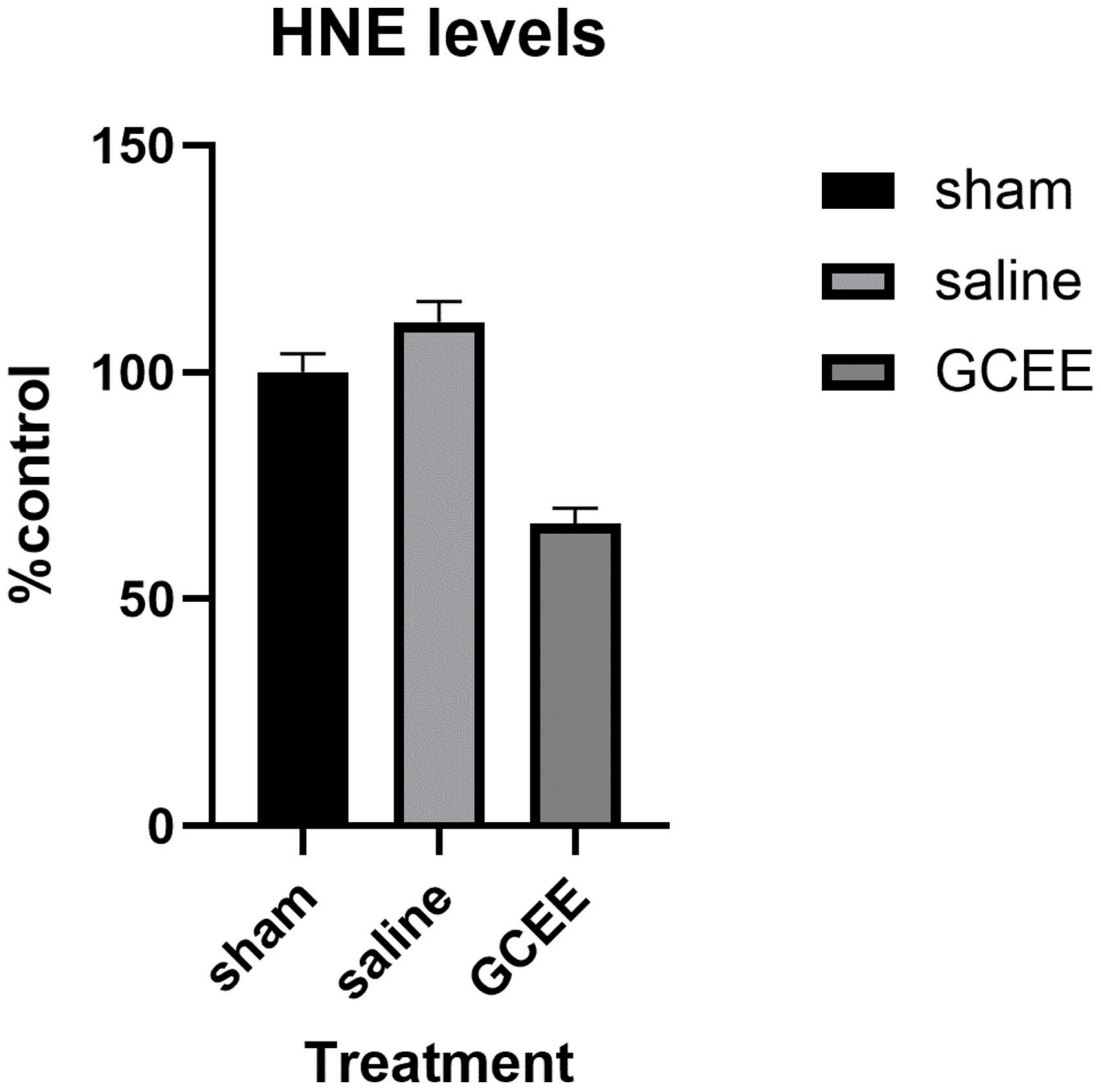
HNE levels in TBI rats administered with GCEE 90 min post injury. Percentage ± SEM. *N* = 6. *p* value >0.05.

## 4 Discussion

Administration with gamma glutamyl cysteinyl ethyl ester at ninety minutes post-injury showed a statistically significant reduction for carbonylated proteins, which was deemed as a successful potential post therapeutic strategy at this time point. The success of GCEE administration at 90 min post-injury suggests GCEE treatment would be helpful if initial injury is sustained at 90 min or less in regard to the protein carbonyl biomarker. Similar results were observed for nitrosative stress as observed in the statistically significant decrease of 3-nitrotyroinse levels. Although levels of HNE were reduced compared to saline, these findings were not statistically significant. The modulation of oxidative stress from this work shows promise of using this antioxidant as a plausible treatment for moderate traumatic brain injury up to ninety minutes post injury, specifically in the reduction of protein carbonyl, HNE, and 3-nitrosine levels. This works bolsters previous findings in which 4-hydroxynonel These findings further support the investigation of expanding the time points of this treatment and mirror findings in regard to the statistically significant declines in protein carbonyl content and 3-nitrotyorsine levels and the non-statistically significant decline of 4-hydroxnonenal levels as a biomarker for lipid peroxidation in previous studies (Henderson et al., 2016; Reed et al., 2009). As a glutathione mimetic, GCEE increases levels of brain glutathione (Drake et al., 2002). This demonstrates an increase in a natural potent endogenous antioxidant in the brain. Other studies support antioxidant elevation to combat oxidative stress via nutritive routes such as but not limited to sulforaphane, riboflavin, polyphenols, tocopherol, and ascorbic acid (Davis and Vemuganti Davis et al., 2022). This work supports elevation of the brain’s most potent antioxidant, glutahione. In conclusion, this study suggests that GCEE can have neuroprotective effects against oxidative stress ninety minutes post-injury. This research is important because the increasing rates and economic impact of traumatic brain injury globally. Moreover, even though there have been years of research on TBI, it has yet to yield a confirmed treatment recommended for traumatic brain injury. GCEE offers promise as a potential postinjury therapeutic for moderate traumatic brain injury at an extended time point.

## Data Availability

The raw data supporting the conclusions of this article will be made available by the authors, without undue reservation.
